# Generic and accurate prediction of retention times in liquid chromatography by post–projection calibration

**DOI:** 10.1038/s42004-024-01135-0

**Published:** 2024-03-08

**Authors:** Yan Zhang, Fei Liu, Xiu Qin Li, Yan Gao, Kang Cong Li, Qing He Zhang

**Affiliations:** 1 Key Laboratory of Groundwater Conservation of MWR, China University of Geosciences, Beijing, 100083 People’s Republic of China; 2 Division of Chemical Metrology and Analytical Science, National Institute of Metrology, Beijing, 100029 People’s Republic of China; 3 Key Laboratory of Chemical Metrology and Applications on Nutrition and Health for State Market Regulation, Beijing, 100029 China

**Keywords:** Mass spectrometry, Cheminformatics, Environmental monitoring, Molecular modelling, Computational chemistry

## Abstract

Retention time predictions from molecule structures in liquid chromatography (LC) are increasingly used in MS–based targeted and untargeted analyses, providing supplementary evidence for molecule annotation and reducing experimental measurements. Nevertheless, different LC setups (e.g., differences in gradient, column, and/or mobile phase) give rise to many prediction models that can only accurately predict retention times for a specific chromatographic method (CM). Here, a generic and accurate method is present to predict retention times across different CMs, by introducing the concept of post–projection calibration. This concept builds on the direct projections of retention times between different CMs and uses 35 external calibrants to eliminate the impact of LC setups on projection accuracy. Results showed that post–projection calibration consistently achieved a median projection error below 3.2% of the elution time. The ranking results of putative candidates reached similar levels among different CMs. This work opens up broad possibilities for coordinating retention times between different laboratories and developing extensive retention databases.

## Abstract (web summary)

Retention time prediction in liquid chromatography plays an important role in molecule annotation and method optimization, however, the accuracy of current quantitative structure−retention relationship models highly depends on the chromatographic method (CM). Here, the authors develop a generic model featuring a post-projection calibration to eliminate the impact of specific CMs, reaching similar ranking levels of putative candidates among different CMs.

## Introduction

Prediction of retention time (RT) in liquid chromatography (LC) has remained an active research field over the last decade^1^, to aid structural identification of unknown molecule^2–4^, rapid chromatographic method (CM) optimization^5,6^, and retention information transfer/harmonization among different laboratories^7–9^. LC coupled to high–resolution mass spectrometry (HRMS) enables high–throughput screening of known and/or unknown molecules at low concentrations, widely applied in environmental and food analysis and in metabolomics^10–13^. Identification workflows in LC–HRMS–based untargeted analysis increasingly include RT prediction steps, as it provides orthogonal evidence for mass spectra to distinguish isobaric molecules^14–16^. By predicting the RTs of putative candidates and comparing them with experimental RT, the number of false–positive candidates can be significantly minimized, thereby reducing experimental measurements and economic costs.

The common strategy for RT prediction employs machine learning algorithms to establish quantitative structure–retention relationships (QSRR). These models rely on extensive RT data from diverse molecular structures to characterize interpretable retention mechanisms and thus make accurate predictions^9,17^. The METLIN database currently stands as one of the largest repositories for such data; however, QSRR models are often constrained by their specificity to particular LC setups due to RT variability among different CMs^18–21^. To enhance the transferability of RT data and predictive models between instruments and CMs—and thus minimize the need for additional experimental measurements—researchers have developed approaches like MultiConditionRT^22^. This QSRR model incorporates molecular descriptors along with CM–specific parameters such as column type and mobile phase composition. Despite these advancements, traditional calibrants may not fully capture complex retention behaviors across different CMs^23–28^. Aalizadeh et al. ^9^ proposed a novel RI system that selects calibrants from a pool of emerging contaminants based on overlapping RTs and chemical similarity indices. This approach aims to provide a more comprehensive elution pattern representation across varying LC setups, achieving median prediction errors ranging from 1.9% to 13.3%.

A further approach for RT prediction is to project known RTs from one CM onto another. The initial strategy involves building a library based on the relationship between the isocratic retention factor (k) and solvent composition (φ), thus estimating RTs for gradient CMs^7,29^. However, this approach requires that the mobile phase, column, and column temperature are the same as those used in the isocratic method, thus limiting the applicability of this strategy. Stanstrup et al. ^8^ introduced a direct RT projection approach that uses known RTs of overlapping molecules on both CMs to yield a non–linear function, i.e., an RT projection model. This model allows for RT transfer between the two CMs, that is, by knowing the RT of a molecule on one CM, the RT of that molecule on the other CM can be predicted. These predictions are extremely accurate over alternative methods, with a median prediction error of <3.7%. For the metabolomics community, such approaches had been used to share the RTs observed in publicly available databases and the RTs predicted by QSRR models with other CMs/laboratories^8,18–21,30^. Predicted–experimental projections can predict the RT of known structure on any CM, while larger prediction errors were expected in contrast to traditional QSRR models and experimental–experimental projections due to the propagation of projection error itself and the inherent prediction error. The accuracy of projections also depended on the overlapping molecules used for model training (i.e., training points)^20,31^ and the similarity in molecule elution order between input and output CMs^8^.

This work presents a strategy to accurately transfer experimental and predicted RTs between different CMs by introducing an external calibration step into projections. Thirty–five molecules were used as calibrants to build a projection model and a reference–projection (ReProjection) model for a given CM (i.e., output CM, OCM). The reference–projected RT (RePRT) derived from the ReProjection model, enables propagating/calibrating the projection error caused by specific LC setups. We focused on the projection errors before and after calibration with different LC setups. The effect of similarity in molecule elution order between input CM (ICM) and reference–input CM (ReICM) and the effect of calibrants on post–projection calibration was analyzed. Further, we tested the ability of post–projection calibration in filtering/ranking putative candidates.

## Results

### Workflow overview

**Fig. 1:**
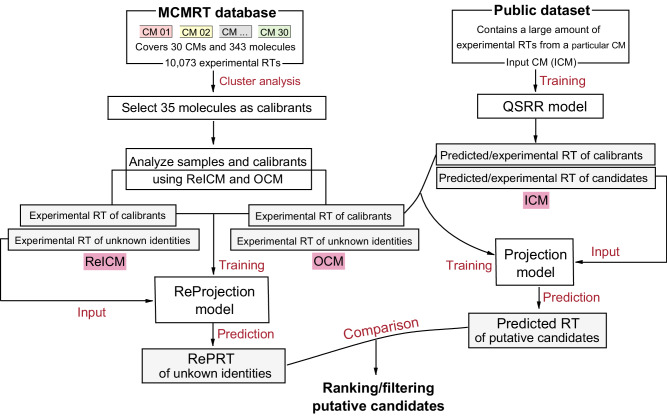
Overview of workflow to transfer experimental/predicted RT data from a publicly available dataset to a given CM. The post–projection calibration approach enhances the accuracy of the transfer.

The objective is to make accurate RT predictions for a given CM using publicly available datasets from other CMs and then use them to improve the confidence of structural annotations. The post–projection calibration method is developed to calibrate the effect of LC setups on RT, which involves first projecting RT from ICM to OCM, and then using a ReICM to externally calibrate the projected RT. The basis for calibrating projection RT is a set of molecules with known RT in ICM, ReICM, and OCM, i.e., calibrants. The workflow is shown schematically in Fig. 1. First of all, an appropriate set of calibrants must be determined to implement RT projection and calibration. Therefore, we developed a multiple CMs–based retention time (MCMRT) database. This database covers 30 CMs with different LC setups and contains >10,000 experimental RTs for 343 molecules with a high diversity of chemical structures. From the database, 35 molecules were selected as calibrants using cluster analysis.

The post–projection calibration method involves three types of CM: (i) CM used in a publicly available dataset, i.e., ICM, (ii) CM used in a local laboratory, i.e., OCM, and (iii) the laboratory–developed CM, used as ReICM, with a similar molecular elution order to the ICM. OCM and ReICM are used for analyzing samples and calibrants. Therefore, in both CMs, the experimental RTs of calibrants and the experimental RTs of unknown identities observed in samples are available. To predict the RT of putative candidates and calibrants not recorded in the public dataset, molecules with known experimental RTs in the dataset are used to train a QSRR model. The RTs of 35 calibrants in ICM and OCM are used to train a main projection model, and the RTs of 35 calibrants in ReICM and OCM are used to train a ReProjection model. The main model projects the experimental/predicted RTs of putative candidates from ICM to OCM, and the ReProjection model projects the experimental RTs of unknown identities from ReICM to OCM. For some candidates, however, there is a large difference between their projected RT and experimental RT due to the differences in retention mechanisms, e.g., different columns, mobile phases, additives, etc. In common cases, they will be filtered incorrectly. This method uses projected RT derived from ReProjection model (i.e., RePRT) instead of experimental RT for unknown identities, and compares it with the projected RT of their putative candidates, which can reduce the number of correct candidates being incorrectly filtered.

### The MCMRT database

**Fig. 2:**
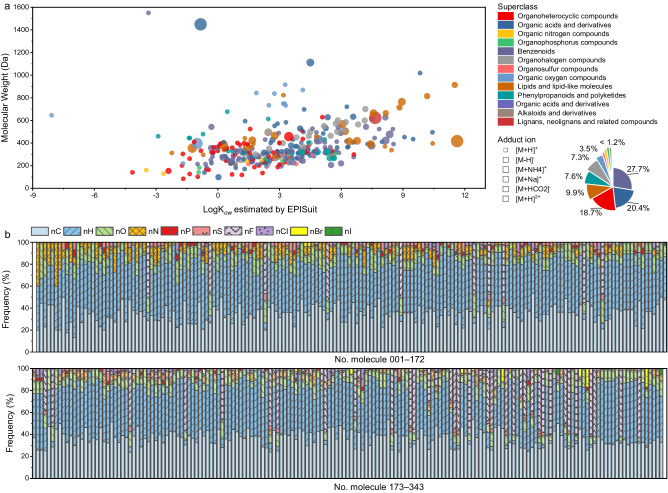
Chemical diversity of molecules in MCMRT. **a** Molecular weight and log *K*_ow_ predicted by EPISuit for each molecule. Each data point corresponds to one molecule in MCMRT; its color indicates the superclass defined by ClassyFire; its size indicates the adduct ion detected by ESI–HRMS. **b** Elemental composition for each molecule. Columns are aligned vertically for each individual molecule.

A total of 343 molecules were selected from various chemical classes and their standard materials were obtained from suppliers. Their RT data was acquired via RPLC/ESI–HRMS using 30 different CMs, respectively (see “Methods” section for details). These molecules covered broad ranges of octanol/water partition coefficient values (log *K*_ow_ − 8.1 to 11.6) and molecular weights (89–1449 Da), enabling to cover the entirety RT range in RPLC and mass–related properties, e.g., including both positive and negative modes and six representative adducts in ESI–MS (Fig. 2a). In terms of chemical classes, they covered 11 ClassyFire’ groups (superclass level)^32^, including benzenoids (27.7%), organic acids and derivatives compounds (20.4%), organoheterocyclic compounds (18.7%), lipids and lipid−like molecules (9.9%), phenylpropanoids and polyketides (7.6%), organohalogen compounds (7.3%), organic oxygen compounds (3.5%), organosulfur compounds (1.2%), organic nitrogen compounds (1.2%), organophosphorus compounds (1.2%), and other compounds (1.5%). Notably, the METLIN database (80,038 molecules) covered seven superclasses, and MCMRT also included these classes, except nucleosides and nucleotides^18^. Furthermore, the organohalogen compounds (e.g., perfluorinated and polyfluoroorganic compounds) and organosulfur compounds contained in MCMRT were not observed in METLIN. Diverse element compositions (C, H, O, N, P, S, Cl, Br, F, and I) indicated that these molecules have a wide range of physiochemical properties (Fig. 2b). These results demonstrated that the molecules in MCMRT are highly diverse and representative of chemical structures. Detailed information, including molecular formula, molecular weight, log *K*_ow_, polarity response, chemical class, etc., can be found in Supplementary Data 1.

The 30 CMs in MCMRT are customized based on common LC setups in untargeted analysis^13,33–36^, covering six C_18_ columns with different specifications (50–150 × 2.1–4.6 mm, 1.7–5 μm), six mobile phase compositions with different buffers (acidic, ammonium, mixed, and semi–mixed), nine running times (10–100 min), seven gradient profiles (single or multiple gradients), five flow rates (constant or variable flow rate, 0.2–1 mL/min), and three column temperatures (30, 40, and 45 °C). Detailed information about the instrumental and chromatographic conditions are described in Table 1 and Supplementary Data 2.

**Table 1:** Chromatographic conditions, source and number of included molecules for datasets used in this study

Dataset	Source	Molecules	Column specifications	Mobile phase A	Mobile phase B	Run Time (min)	LC gradient (t[min], flow rate[mL/min], %B)	Column tem. (°C)
CM 01	MCMRT	335	ACQUITY PRIMER HSS T3 (100 × 2.1 mm, 1.8 μm)	90% H2O + 10% MeOH + 0.01% FA + 5 mM NH4COOH	MeOH + 0.01% FA + 5 mM NH4COOH	10	(0, 0.2, 5), (1, 0.2, 5), (2, 0.2, 40), (6, 0.2, 100), (8, 0.2, 100), (8.1, 0.2, 5), (10, 0.2, 5)	30
CM 02	MCMRT	335	Acclaim RSLC 120 C18 (100 × 2.1 mm, 2.2 μm)	90% H2O + 10% MeOH + 0.01% FA + 5 mM NH4COOH	MeOH + 0.01% FA + 5 mM NH4COOH	15	(0, 0.3, 0), (2, 0.3, 0), (10, 0.3, 100), (12, 0.3, 100), (12.1, 0.3, 0), (15.1, 0.3, 0)	30
CM 03	MCMRT	335	Acclaim RSLC 120 C18 (100 × 2.1 mm, 2.2 μm)	90% H2O + 10% MeOH + 0.01% FA + 5 mM NH4COOH	MeOH + 0.01% FA + 5 mM NH4COOH	21	(0, 0.2, 1), (1, 0.2, 1), (3, 0.2, 39), (14, 0.4, 99), (16, 0.48, 99), (16.1, 0.2, 1), (21, 0.2, 1)	30
CM 04	MCMRT	335	Thermo Hypersil GOLD (100 × 2.1 mm, 1.9 μm)	90% H2O + 10% MeOH + 0.01% FA + 5 mM NH4COOH	MeOH + 0.01% FA + 5 mM NH4COOH	21	(0, 0.2, 1), (1, 0.2, 1), (3, 0.2, 39), (14, 0.4, 99), (16, 0.48, 99), (16.1, 0.2, 1), (21, 0.2, 1)	30
CM 05	MCMRT	335	ACQUITY BEH C18 (100 × 2.1 mm, 1.7 μm)	90% H2O + 10% MeOH + 0.01% FA + 5 mM NH4COOH	MeOH + 0.01% FA + 5 mM NH4COOH	21	(0, 0.2, 1), (1, 0.2, 1), (3, 0.2, 39), (14, 0.4, 99), (16, 0.48, 99), (16.1, 0.2, 1), (21, 0.2, 1)	40
CM 06	MCMRT	335	ACQUITY PRIMER HSS T3 (100 × 2.1 mm, 1.8 μm)	90% H2O + 10% MeOH + 0.01% FA + 5 mM NH4COOH	MeOH + 0.01% FA + 5 mM NH4COOH	21	(0, 0.2, 0), (2, 0.2, 0), (4, 0.2, 40), (15, 0.2, 100), (18, 0.2, 100), (18.1, 0.2, 0), (21, 0.2, 0)	30
CM 07	MCMRT	335	Acclaim RSLC 120 C18 (100 × 2.1 mm, 2.2 μm)	90% H2O + 10% MeOH + 0.01% FA + 5 mM NH4COOH	MeOH + 0.01% FA + 5 mM NH4COOH	30	(0, 0.2, 1), (1, 0.2, 1), (5, 0.2, 39), (21, 0.4, 99), (25 0.48, 99), (25.1, 0.2, 1), (30, 0.2, 1)	30
CM 08	MCMRT	335	Acclaim RSLC 120 C18 (100 × 2.1 mm, 2.2 μm)	90% H2O + 10% MeOH + 0.01% FA + 5 mM NH4COOH	MeOH + 0.01% FA + 5 mM NH4COOH	30	(0, 0.3, 0), (2, 0.3, 0), (24, 0.3, 100), (27, 0.3, 100), (27.1, 0.3, 0), (15.1, 0.3, 0)	30
CM 09	MCMRT	335	Thermo Hypersil GOLD (100 × 2.1 mm, 1.9 μm)	90% H2O + 10% MeOH + 0.01% FA + 5 mM NH4COOH	MeOH + 0.01% FA + 5 mM NH4COOH	30	(0, 0.3, 0), (2, 0.3, 0), (24, 0.3, 100), (27, 0.3, 100), (27.1, 0.3, 0), (15.1, 0.3, 0)	30
CM 10	MCMRT	335	Acclaim RSLC 120 C18 (100 × 2.1 mm, 2.2 μm)	90% H2O + 10% MeOH + 0.01% FA + 5 mM NH4COOH	MeOH + 0.01% FA + 5 mM NH4COOH	30	(0, 0.2, 0), (2, 0.2, 0), (24, 0.2, 100), (27, 0.2, 100), (27.1, 0.2, 0), (15.1, 0.2, 0)	30
CM 11	MCMRT	335	ACQUITY BEH C18 (100 × 2.1 mm, 1.7 μm)	90% H2O + 10% MeOH + 0.01% FA + 5 mM NH4COOH	MeOH + 0.01% FA + 5 mM NH4COOH	30	(0, 0.2, 0), (2, 0.2, 0), (24, 0.2, 100), (27, 0.2, 100), (27.1, 0.2, 0), (15.1, 0.2, 0)	30
CM 12	MCMRT	335	Acclaim RSLC 120 C18 (100 × 2.1 mm, 2.2 μm)	90% H2O + 10% MeOH + 0.01% FA + 5 mM NH4COOH	MeOH + 0.01% FA + 5 mM NH4COOH	45	(0, 0.3, 0), (2, 0.3, 0), (38, 0.3, 100), (42, 0.3, 100), (42.1, 0.3, 0), (45.1, 0.3, 0)	30
CM 13	MCMRT	335	ACQUITY PRIMER HSS T3 (100 × 2.1 mm, 1.8 μm)	90% H2O + 10% MeOH + 0.01% FA + 5 mM NH4COOH	MeOH + 0.01% FA + 5 mM NH4COOH	60	(0, 0.2, 1), (1, 0.2, 1), (12, 0.2, 40), (40, 0.2, 100), (49, 0.2, 100), (50, 0.2, 1), (60, 0.2, 1)	30
CM 14	MCMRT	335	ACQUITY UPLC HSS T3 (2.1 ×50 mm,1.8 μm)	90% H2O + 10% MeOH + 0.01% FA + 5 mM NH4COOH	MeOH + 0.01% FA + 5 mM NH4COOH	60	(0, 0.2, 1), (1, 0.2, 1), (12, 0.2, 40), (40, 0.2, 100), (49, 0.2, 100), (50, 0.2, 1), (60, 0.2, 1)	30
CM 15	MCMRT	335	Acclaim 120 C18 (4.6 ×150 mm, 5 μm)	90% H2O + 10% MeOH + 0.01% FA + 5 mM NH4COOH	MeOH + 0.01% FA + 5 mM NH4COOH	60	(0, 1, 5), (1, 1, 5), (12, 1, 40), (40, 1, 100), (49, 1, 100), (50, 1, 5), (60, 1, 5)	30
CM 16	MCMRT	335	ACQUITY PRIMER HSS T3 (100 × 2.1 mm, 1.8 μm)	90% H2O + 10% MeOH + 0.01% FA + 5 mM NH4COOH	MeOH + 0.01% FA + 5 mM NH4COOH	100	(0, 0.2, 1), (2, 0.2, 1), (40, 0.2, 40), (70, 0.2, 100), (89, 0.2, 100), (90, 0.2, 1), (100, 0.2, 1)	30
CM 17	MCMRT	335	Thermo Hypersil GOLD (100 × 2.1 mm, 1.9 μm)	H2O + 0.1% FA + 4 mM NH4COOH	MeOH + 0.1% FA + 4 mM NH4COOH	14	(0, 0.3,12), (8, 0.3, 95), (9, 0.3, 100), (11, 0.3, 100), (11.1, 0.3, 12), (14, 0.3, 12)	45
CM 18	MCMRT	335	Thermo Hypersil GOLD (100 × 2.1 mm, 1.9 μm)	H2O + 0.1% FA + 4 mM NH4COOH	MeOH + 0.1% FA + 4 mM NH4COOH	21	(0, 0.2, 1), (1, 0.2, 1), (3, 0.2, 39), (14, 0.4, 99), (16, 0.48, 99), (16.1, 0.2, 1), (21, 0.2, 1)	30
CM 19	MCMRT	335	Acclaim RSLC 120 C18 (100 × 2.1 mm, 2.2 μm)	90% H2O + 10% MeOH + 0.01% FA + 5 mM NH4COOH	ACN	21	(0, 0.2, 1), (1, 0.2, 1), (3, 0.2, 39), (14, 0.4, 99), (16, 0.48, 99), (16.1, 0.2, 1), (21, 0.2, 1)	30
CM 20	MCMRT	330	ACQUITY PRIMER HSS T3 (100 × 2.1 mm, 1.8 μm)	H2O + 0.1% FA	ACN + 0.1% FA	20	(0, 0.5, 2), (2, 0.5, 2), (15, 0.5, 99), (17, 0.5, 99), (18, 0.5, 2), (20, 0.5, 2)	30
CM 21	MCMRT	330	Thermo Hypersil GOLD (100 × 2.1 mm, 1.9 μm)	H2O + 0.1% FA	ACN + 0.1% FA	21	(0, 0.2, 1), (1, 0.2, 1), (3, 0.2, 39), (14, 0.4, 99), (16, 0.48, 99), (16.1, 0.2, 1), (21, 0.2, 1)	30
CM 22	MCMRT	330	ACQUITY PRIMER HSS T3 (100 × 2.1 mm, 1.8 μm)	H2O + 0.1% FA	ACN + 0.1% FA	45	(0, 0.2, 5), (2, 0.2, 5), (10, 0.2, 40), (35, 0.2, 100), (39, 0.2, 100), (40, 0.2, 5), (45, 0.2, 5)	30
CM 23	MCMRT	330	ACQUITY UPLC HSS T3 (2.1 × 50 mm,1.8 μm)	H2O + 0.1% FA	ACN + 0.1% FA	45	(0, 0.2, 5), (2, 0.2, 5), (10, 0.2, 40), (35, 0.2, 100), (39, 0.2, 100), (40, 0.2, 5), (45, 0.2, 5)	30
CM 24	MCMRT	330	Acclaim 120 C18 (4.6 × 150 mm, 5 μm)	H2O + 0.1% FA	ACN + 0.1% FA	45	(0, 1, 5), (2, 1, 5), (10, 1, 40), (35, 1, 100), (39, 1, 100), (40, 1, 5), (45, 1, 5)	30
CM 25	MCMRT	343	ACQUITY PRIMER HSS T3 (100 × 2.1 mm, 1.8 μm)	H2O + 5 mM NH4CH2COOH	ACN + 5 mM NH4CH2COOH	20	(0, 0.2, 5), (1, 0.2, 5), (7, 0.2, 40), (14, 0.2, 100), (18, 0.2, 100), (18.1, 0.2, 5), (20, 0.2, 5)	30
CM 26	MCMRT	343	ACQUITY UPLC HSS T3 (2.1 × 50 mm,1.8 μm)	H2O + 5 mM NH4CH2COOH	ACN + 5 mM NH4CH2COOH	20	(0, 0.2, 5), (1, 0.2, 5), (7, 0.2, 40), (14, 0.2, 100), (18, 0.2, 100), (18.1, 0.2, 5), (20, 0.2, 5)	30
CM 27	MCMRT	343	ACQUITY PRIMER HSS T3 (100 × 2.1 mm, 1.8 μm)	H2O + 5 mM NH4CH2COOH	ACN + 5 mM NH4CH2COOH	20	(0, 0.2, 5), (2, 0.2, 5), (6, 0.2, 40), (20, 0.2, 100), (25, 0.2, 100), 26, 0.2, 5), (30, 0.2, 5)	30
CM 28	MCMRT	343	ACQUITY UPLC HSS T3 (2.1 × 50 mm,1.8 μm)	H2O + 5 mM NH4CH2COOH	ACN + 5 mM NH4CH2COOH	30	(0, 0.2, 5), (2, 0.2, 5), (6, 0.2, 40), (20, 0.2, 100), (25, 0.2, 100), 26, 0.2, 5), (30, 0.2, 5)	30
CM 29	MCMRT	343	Acclaim 120 C18 (4.6 × 150 mm, 5 μm)	H2O + 5 mM NH4CH2COOH	ACN + 5 mM NH4CH2COOH	30	(0, 1, 5), (2, 1, 5), (6, 1, 40), (20, 1, 100), (25, 1, 100), 26, 1, 5), (30, 1, 5)	30
CM 30	MCMRT	343	Acclaim RSLC 120 C18 (100 × 2.1 mm, 2.2 μm)	H2O + 5 mM NH4COOH	ACN + 5 mM NH4COOH	21	(0, 0.2, 1), (1, 0.2, 1), (3, 0.2, 39), (14, 0.4, 99), (16, 0.48, 99), (16.1, 0.2, 1), (21, 0.2, 1)	30
CM 03p	Aalizadeh, et al. ^9^	1820	Acclaim RSLC 120 C18 (100 × 2.1 mm, 2.2 μm)	90% H2O + 10% MeOH + 0.01% FA + 5 mM NH4COOH	MeOH + 0.01% FA + 5 mM NH4COOH	21	(0, 0.2, 1), (1, 0.2, 1), (3, 0.2, 39), (14, 0.4, 99), (16, 0.48, 99), (16.1, 0.2, 1), (21, 0.2, 1)	30

As a result, a total of 10,073 experimental RT values from 30 CMs were included in the MCMRT, of which 330 molecules had experimental RT values on all CMs, i.e., they overlapped between these CMs. These experimental RT values are available in Supplementary Data 3. It is worthing noting that eight environmental estrogens were not detected in CMs 01–24 containing acidic additives, and 5 other benzenoids and lipid–like molecules were not detected in ammonium–freed CMs 20–24 due to the lack of their preferred adducts (i.e., [M + NH4]+). The experimental RTs of all molecules in MCMRT were evenly distributed within each CM running time, indicating that these molecules can demonstrate the entirety RT range in LC and have no obvious preference for specific RT ranges.

### Retention behavior classification and calibrants selection

**Fig. 3:**
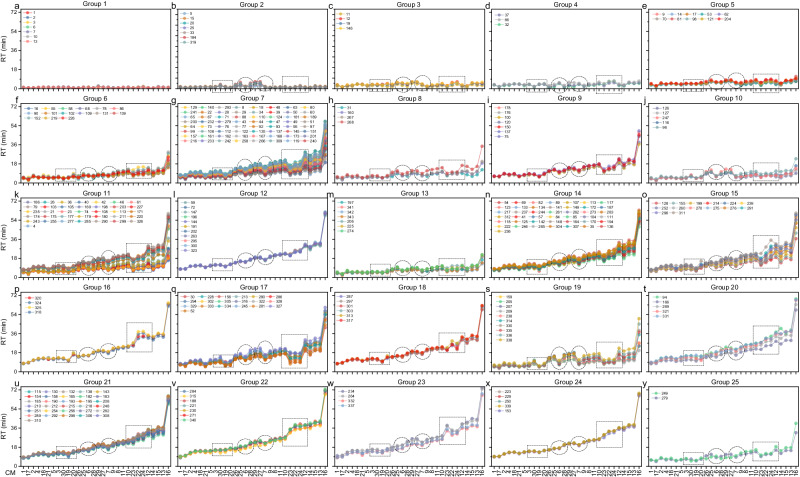
Experimental RTs of 330 overlapping molecules in 30 different CMs. These molecules are divided into 25 groups based on their retention behavior (**a**–**y**). Each RT profile corresponds to one molecule in MCMRT.

Thirty RT datasets in MCMRT were analyzed for 330 overlapping molecules using self–organizing mapping (SOM) clustering algorithm^37,38^, to characterize their retention behavior. These molecules were classified into 25 groups, each exhibited a specific RT distribution and similar retention behavior with different LC setups (Fig. 3 and Supplementary Data 3). Specifically, non–retained molecules fell into groups 1 and 2, with group 2 molecules showing enhanced retention in CMs lacking acidic additives (CMs 25–30). Meanwhile, groups 3 to 5 exhibited weaker retention across all CMs, in contrast to groups 12, 16, 21–24, which showed notably stronger retention. Groups 7, 9, 11, and 14 demonstrated similar retention patterns, representing a general variation in RT between different LC setups. Large differences were observed in other groups, such as group 15 molecules having longer RT in CM 24 than in CM 22, while group 18 showed the opposite trend, and some groups exhibited almost identical RTs across certain CMs. These results demonstrated that although in most cases many molecules exhibit consistent RT changes between two different CMs, there can still be several outgeneral changes for a considerable number of molecules.

Five different sets of calibrants (A–E) were selected from the 330 molecules. The aim was to select the most appropriate set of calibrants for reliable post–projection calibrations. These sets ranged in size from 27 to 72 molecules and covered up to 25 retention behaviors (Supplementary Data 4). Set E was specially customized to cover only 17 retention behaviors. As the number of calibrants increased within a set, a broader spectrum of general retention behaviors was encompassed. The correlation analysis conducted between experimental RTs across different CMs, utilizing these calibrant sets and the 330 molecules (Fig. S1 and Supplementary Data 5). For sets A, B, C, and D, the r2 difference between calibrants and 330 molecules was in all cases below 0.07, while for set E it can be as high as 0.16. In addition, calibrants from sets A, B, C, and D not only covered the entire RT range of each CM, but also exhibited RT profile highly similar to the 330 molecules (Figs. S2–5). Although calibrants in set E covered the entire RT range, they exhibited limited representativeness for molecules with outgeneral retention behavior (Fig. S6). Collectively, calibrant sets A, B, C, and D can account for the effect of LC setups on molecular elution order and demonstrate the overall elution pattern of a CM. For further details on this section, refer to the Supplementary discussions.

### Post–projection calibration and performance validation

**Fig. 4:**
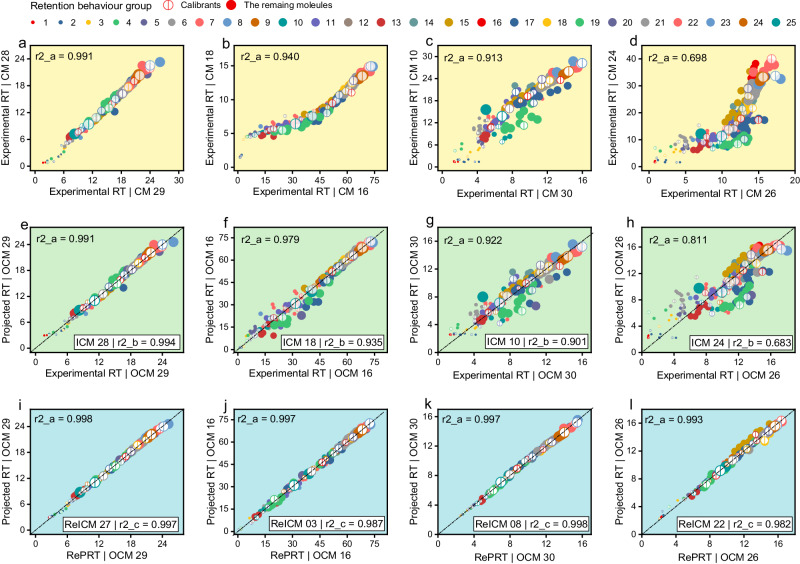
Examples of projection models between the RTs of molecules in two different CMs. Examples are given of a “good” model for projecting experimental RTs from ICM 28 to OCM 29 (**e**), a model with a few outliers for projecting experimental RTs from ICM 18 to OCM 16 (**f**), a model with several outliers for projecting experimental RTs from ICM 10 to OCM 30 (**g**), and a model with many outliers for projecting experimental RTs from ICM 24 to OCM 26 (**h**). Good calibrations for these projections are given using CM 27 (**i**), CM 03 (**j**), CM 08 (**k**), and CM 22 (**l**) as ReICM for ICMs 28, 07, 10 and 22, respectively. Panels **a**–**d** show the relation between the experimental RTs in two different CMs before projection. Panels **e**–**h** show the relation between experimental and projected RTs in OCM before calibration. Panels **i**–**l** show the relation between projected and reference–projected RTs in OCM after calibration. r2_a indicates square of correlation coefficient between RTs for all molecules in MCMRT. r2_b indicates square of correlation coefficient between the experimental RTs in ICM and OCM for 35 calibrants in set B. r2_c indicates square of correlation coefficient between the experimental RTs in ICM and ReICM for 35 calibrants in set B. Its color and size indicate the group classified based on their retention behavior (a total of 25 groups). Points labeled with hollow circle indicate the calibrants, while that labeled with solid circle indicate the remaining molecules.

We validated the performance of post–projection calibration using 30 different CMs in MCMRT. Specifically, all CMs were used as ICMs, and for each ICM, an appropriate ReICM was selected from the remaining 29 CMs, followed by using the remaining 28 CMs as their OCMs, respectively. To select the most appropriate calibrants, we used five different calibrant sets (A–E) to perform post–projection calibration, including projecting all experimental RTs from ICM to OCM and calibrating all projected RTs in each OCM (see “Methods” section for details). To investigate the impact of CM similarity on post–projection calibration, we categorized all OCMs into four groups based on the differences in LC setups: (1) OCMA, having the same mobile phase composition as ICM; (2) OCMsB, having a mobile phase composition distinct but similar to ICM; (3) OCMsC, having a slightly different mobile phase composition from ICM; and (4) OCMsD, having a largely different mobile phase composition from ICM. See Supplementary Data 6 for details on all OCM groupings. Illustrations of the relationships between experimental RTs in the ICM and each group of OCMs—OCMsA (Fig. 4a), OCMsB (Fig. 4b), OCMsC (Fig. 4c), and OCMsD (Fig. 4d)—are provided. The r2 values between experimental RTs of all molecules were 0.892–0.998, 0.906–0.993, 0.798–0.947, and 0.698–0.860 for OCMsA, OCMsB, OCMsC, and OCMsD, respectively (Fig. S7).

**Fig. 5:**
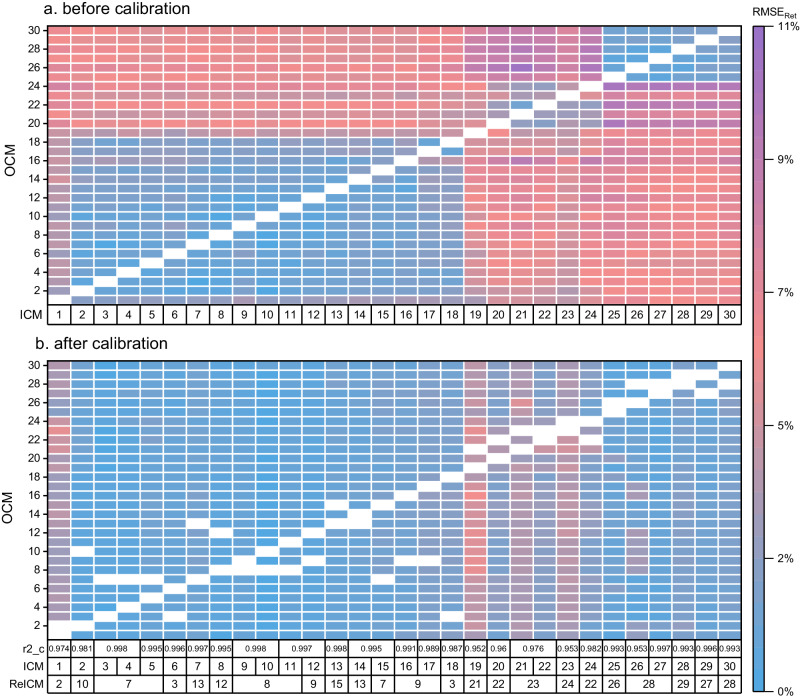
Experimental–experimental projection results before and after calibration. Panels **a**, **b** show the effect of different ICMs, OCMs and ReICMs on experimental–experimental projections before calibration (**a**) and after calibration (**b**). Set B was used as calibrants. The RMSE_Ret_ of all projections between each pair of CMs was used as assessment index.

**Fig. 6:**
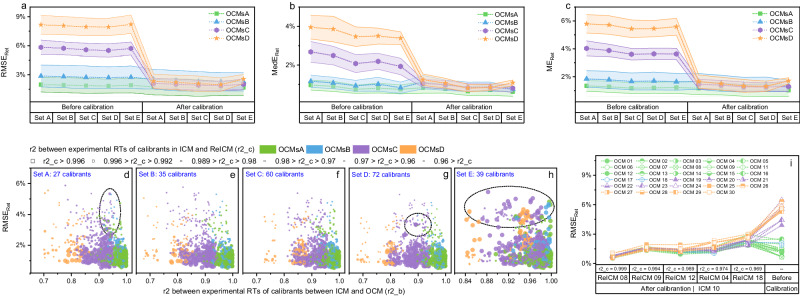
The effect of five different sets of calibrants on experimental–experimental projections before and after calibration. Panels **a**–**c** show the effect of five different sets of calibrants on experimental–experimental projections before and after calibration, using RMSE_Ret_ (**a**), MedE_Ret_ (**b**) and ME_Ret_ (**c**) as assessment indexes, respectively. The error bands represent the standard deviation of all corresponding values in each group (n_OCMsA_ = 83,160, n_OCMsB_ = 27,720, n_OCMsC_ = 142,560, n_OCMsD_ = 23,760). Panels **d**–**h** show the relation between the RMSE_Ret_ after calibration, the square of correlation coefficient between the calibrant experimental RTs in ICM and OCM (r2_b), and the square of correlation coefficient between the calibrant experimental RTs in ICM and ReICM (r2_c), using calibrant set A (**d**), set B (**e**), set C (**f**), Set D (**g**), and Set E (**h**), respectively. Panel **i** shows the effect of different ReICMs on post–projection calibrations. Set B was used as calibrants.

First, we compared the performance of experimental–experimental projections using different ICM and OCM pairs. Since the relative projection error for non–retained and weakly retained molecules exceeded 500% in some cases, the error relative to elution time (ERet) was used for comparison. Elution time refers to the maximum RT of calibrants in OCM. Figure 5a and Supplementary Data 7 show the projection results for each ICM and OCM pair using set B as calibrants. These errors varied across different OCM groups. Taken projections of experimental RT from ICM 25 to the remaining 29 OCMs as examples. The root mean square error relative to elution time (RMSERet) was between 0.9 and 1.8% (0.2 and 0.4 min) in OCMsA and B, while that was between 5.4 and 9.9% (0.6 and 5.5 min) in OCMsC and D. Similar results were observed for the remaining 29 ICMs. For all projection errors across the same OCM group, the RMSERet values were 1.9, 2.8, 5.7, and 8.1% for OCMsA, B, C, and D, respectively (Fig. 6a). In minutes, their RMSE was 0.4, 0.6, 1.7, and 1.9 min, respectively. These results demonstrated that the CM similarity between ICM and OCM determines the projection accuracy. Collectively, experimental–experimental projections between two CMs with the same mobile phase composition allows the projection of RTs with extremely high accuracy (RMSE_Ret_ < 2.6%). However, large differences in elution time (e.g., RMSE_Ret_ = 4.3% between ICM 01 and OCM 16), and LC column (e.g., RMSE_Ret_ = 5.0% between ICM 24 and OCM 23) increased projection errors. Yet, different mobile phase compositions further declined the accuracy of projections^31^, with RMSE_Ret_ as high as 10.3%. To the best of our knowledge, projection errors of this magnitude are often neglected.

Next, we demonstrated that post–projection calibration method minimized the effect of LC setups on projection accuracy. Examples were given of a “good” model for projecting experimental RTs to OCMsA (Fig. 4e), a model with a few outliers for projecting experimental RTs to OCMsB (Fig. 4f), a model with several outliers for projecting experimental RTs to OCMsC (Fig. 4g), and a model with many outliers for projecting experimental RTs to OCMsD (Fig. 4h). The r2 between projected and experimental RT values for these four models were 0.991, 0.979, 0.922, and 0.811, respectively. It can be seen that in OCMsB, C, and D, the molecules in groups 17 and 19 had large projection errors. In particular, in OCMsD, almost all molecules had larger projection errors except those molecules with general retention behavior. Using RePRT instead of experimental RT, these projections were well calibrated (Fig. 4i–l). In all cases, the r2 between projected RT and RePRT was >0.993. Figure 5b and Supplementary Data 8 show the calibration results for each ICM and OCM pair. For all calibration errors across the same OCM group, the RMSE_Ret_ values were 1.7, 2.4, 2.0, and 2.2% for CMsA, B, C, and D, respectively (Fig. 6a). Compared with the results before calibration, these RMSE_Ret_ values decreased by 10.2, 14.4, 64.4, and 72.6%, respectively. Therefore, the results confirmed that post–projection calibration method can accurately transfer RT across a wider range of CMs.

We also demonstrated that the CM similarity between ICM and ReICM determines the calibration accuracy. To validate this, CMs 04, 08, 09, 12, and 18 were used as ReICMs for ICM 10, respectively. The r2 (i.e., r2_c) between experimental RTs of calibrants in ICM and ReICM was used to determine the CM similarity. Results from these calibrations are shown in Fig. 6i and Supplementary Data 9. It can be seen that before calibration, the RMSE_Ret_ across all projections in an OCM ranged from 0.6% to 6.5%, whereas after calibration using ReICM 08 (r2_c = 0.999), this RMSE_Ret_ ranged from 0.5% to 1.1%. Yet, the RMSE_Ret_ increased with decreasing r2_c, e.g., changing ReICM 08 to ReICM 18 (r2_c = 0.969) increased the RMSE_Ret_ from below 1.1% to above 1.8%. Consistent conclusions were also drawn from other ICMs that the calibration accuracy decreased with decreasing CM similarity between ICM and ReICM (Figs. 5b and  6d–h).

Finally, we compared the performance of post–projection calibration using five different sets of calibrants. Results from these projections and calibrations can be found in Supplementary Data 10–11. As the number of calibrants increased from 27 (set A) to 72 (set D), there were no large difference in RMSE_Ret_ values (Fig. 6a), a slight decrease in mean error relative to elution time (ME_Ret_) values (Fig. 6b), and a large decrease in median error relative to elution time (MedE_Ret_) values (Fig. 6c). Although set E contains 39 calibrants, it yielded the smallest MedE_Ret_ values compared to the other four sets, especially for projection errors in OCMsC and D. Nevertheless, for calibration errors in an OCM, the minimum RMSE_Ret_, MedE_Ret_ and ME_Ret_ values were often derived from set C or set D, while for calibration errors in OCMsC and D, set E can yield the maximum RMSE_Ret_, MedE_Ret_ and ME_Ret_ values. Because the number of calibrants with general retention behavior in set B was less than that in set C, the calibration accuracy using set B was lower than that using set C. Fortunately, the calibrations using sets B and C had consistent accuracy when r2_c higher than 0.992. In addition, a larger number of calibrants with outgeneral retention behavior in set D may affect the fitting of projection models. These results demonstrated that the number of calibrants and the retention behavior of calibrants both determines the accuracy of post–projection calibration.

We also observed that for sets A, B, C, and D, the projection error correlated with the r2 (r2_b) between experimental RTs of calibrants in ICM and OCM. Specifically, the r2 between RMSE_Ret_ and r2_b ranged from 0.561 to 0.734, while for set E, it was only 0.199 (Fig. S8). Furthermore, when r2_c was higher than 0.992 using sets B and C (Fig. 6e, f), the RMSE_Ret_ values were in all cases below 3.0%. However, these RMSE_Ret_ values were as high as 5.5% when using set E, even though r2_c was higher than 0.996 (Fig. 6h). These results demonstrated that projection accuracy can be estimated from r2_b and calibration accuracy can be estimated from r2_c using sets B and C. Nevertheless, this estimation is no longer valid if set E is used. The main reason is that the calibrants in set E had limited retention behaviors, which cannot account for the effect of LC setups on the RT profile of a CM. In order to reduce experimental costs, 35 molecules in set B were finally accepted as calibrants.

### Application of post–projection calibration for predicted RT

To explore the scalability of post–projection calibration, we validated the performance of projecting predicted RT onto different CMs and assessed how well projected RT could annotate unknown identities. First, we employed a robust QSRR model, constructed using a publicly accessible dataset^9^ (see Methods for details). The dataset contains experimental RTs for 1820 emerging contaminants, which were measured in a specific CM (referred to as CM 03p). For model training, we randomly selected 75% of the molecules from CM 03p—excluding 154 that overlapped with our set of 343 molecules—to create a training set of 1,258 molecules. The remaining molecules, including those overlapping, were allocated to the validation set (*N* = 562). Next, we selected 112 molecules from MCMRT as unknown identities, characterized by monoisotopic masses ranging from 119 to 1449 Da, log *K*_ow_ values spanning from −8.1 to 10.4, and encompassing 20 distinct retention behaviors. To mimic scenarios commonly encountered in untargeted analysis—where a feature peak is detected after data processing while its identity remains unknown—we compiled a list of 2935 putative candidates (3–91 in each). This list included 1945 isomeric candidates sourced from the ChemSpider and PubChem websites and an additional 990 isobaric candidates (with a mass error tolerance of 10 ppm) derived from the METLIN database. We then employed the QSRR model to predict RTs for both the non–overlapping set of 199 molecules in MCMRT and the entire pool of putative candidates. Next, the QSRR model was used to predict RT for 199 non–overlapping molecules in MCMRT and 2935 putative candidates. These predictions were projected from CM 03p onto our suite of 30 CMs using predicted–experimental projections—with the exception of CM 07 which was used as the ReICM (see Methods for details).

**Fig. 7:**
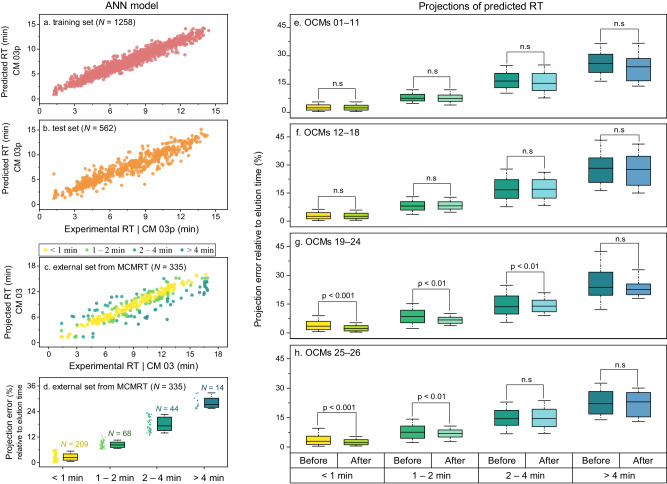
QSRR model and predicted-experimental projection results before and after calibration. **a**, **b** Correlations between experimental and predicted RTs for molecules in training (**a**) and validation sets (**b**) for QSRR model. **c** Correlation between experimental and projected RTs for molecules in MCMRT (external set). **d** Projection error relative to elution time for molecules with different prediction error. Panels **e**–**h** show the differences between predicted–experimental projection results before and after calibration for OCMs 01–11 (**e**), OCMs 12–18 (**f**), OCMs 19–24 (**g**), and OCMs 25–30 (**h**), respectively. All projections and calibrations from the 30 CMs (excluding ReICM 07) were used for calculation; the box plot represents median value (line) and interquartile range (25–75% percentiles) excluding outliers; the error bands represent 10–90% percentiles; *P* values obtained from t–tests are provided (*n*_< 1 min_ = 2090, *n*_1-2 min_ = 680, *n*_2-4 min_ = 440, *n*_> 4 min_ = 140 in OCMs 01–11; *n*_< 1 min_ = 1463, *n*_1-2 min_ = 476, *n*_2-4 min_ = 308, *n*_> 4 min_ = 98 in OCMs 12–18; *n*_< 1 min_ = 1231, *n*_1-2 min_ = 408, *n*_2-4 min_ = 264, *n*_> 4 min_ = 84 in OCMs 19–24; *n*_< 1 min_ = 1254, *n*_1-2 min_ = 408, *n*_2-4 min_ = 264, *n*_> 4 min_ = 84 in OCMs 25–30) for a 95% confidence interval.

Prediction results of the QSRR model for the training and validation sets are shown in Fig. 7a–b and Supplementary Data 12. The RMSE_Ret_ values were 4.9% and 7.3%, while r2 were 0.943 and 0.885, respectively. In parallel studies utilizing the same dataset, Aalizadeh et al. ^17^ employed a support vector machine approach for QSRR modeling and achieved comparable results to our model, reporting RMSE_Ret_ values of 5.4% for the training set and 8.3% for the validation set. From all the molecules in MCMRT, 335 with known experimental RT in CM 03 were used as an external set to assess the model’s generalization ability. We assessed the accuracy of our model by comparing projected RT against experimental RT for these molecules in CM 03, obtaining RMSE_Ret_ and r2 values of 10.0% and 0.821, respectively, as shown in Fig. 7c. Although ICM 03p and OCM 03 were highly similar in LC setups, these projection errors varied depending on the molecular structure. Specifically, most antibiotics, pharmaceuticals and pesticides had projection errors smaller than 1 min, while perfluorinated compounds, organophosphorus flame retardants and mycotoxins usually had projection errors greater than 2 min. This is due to the fact that molecules structurally similar to the former were available in the public dataset, whereas that to the latter cannot be found in this dataset. These results demonstrated the structural similarity between the training dataset and the input chemical structure affected the accuracy of predicted RT^18^, and thereby affecting the accuracy of predicted–experimental projections.

Projection and calibration results for the external set are shown in Fig. 7e–h and Supplementary Data 13. The molecules in the external set were divided into four groups based on their projection error in CM 03. Among them, group 1 contains 209 molecules with an error of less than 1 min, group 2 contains 68 molecules with an error of between 1 and 2 min, group 3 contains 44 molecules with an error of between 2 and 4 min, and group 4 contains 14 molecules with an error of greater than 4 min (Fig. 5d). For all OCMs, the projection results of groups 1 and 2 (RMSE_Ret_ < 11.0%) were largely smaller than that of groups 3 and 4 (RMSE_Ret_ > 14.6%). Similar results were also observed for their calibration errors; the mean absolute error (MAE) spanned from 0.7 min and 2.0 min in groups 1 and 2 to 4.2 min and 6.7 min in groups 3 and 4, respectively. Furthermore, for the molecules in groups 1 and 2, the MAE in OCMs 01–11, OCMs 12–18, OCMs 19–24 and OCMs 25–30 was reduced by 0.1%, -2.2%, 25.2% and 18.9% after calibration. These results demonstrated that for molecules with small prediction errors, the projection results after calibration were significantly better than those before calibration in an OCM where the mobile phase composition is different from ICM03p (T–test, *P* < 0.001, *n*_< 1 min_ = 1231 and *n*_1-2 min_ = 408 in OCMs 19–24, Fig. 7g; T–test, *P* < 0.001, *n*_< 1 min_ = 1254 and *n*_1-2 min_ = 408 in OCMs 25–30, Fig. 7h). Yet, no statistically significant differences in accuracy (mean error) were generally observed for molecules with large prediction errors (T–test, *P* > 0.05, *n*_2-4 min_ = 308 in OCMs 12–18, *n*_2-4 min_ = 264 in OCMs 19–24, *n*_2-4 min_ = 264 in OCMs 25–30, Fig. 7f–h).

**Fig. 8:**
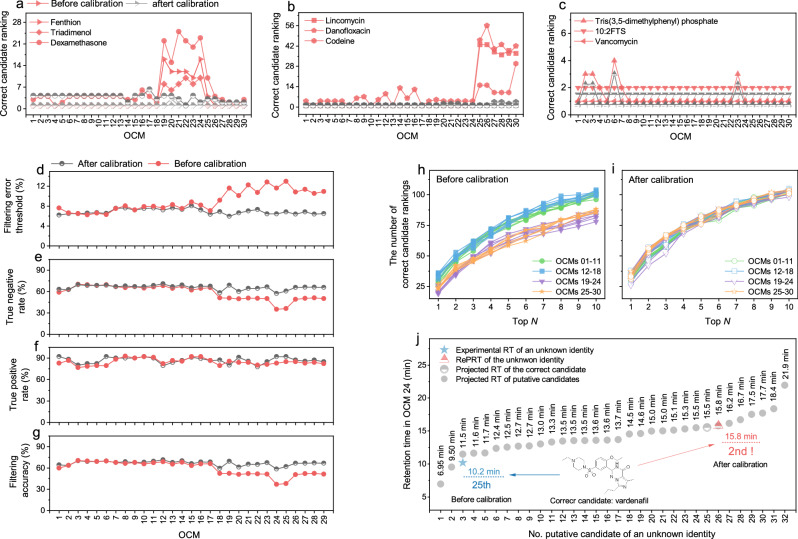
Post–projection calibration improves unknown identities annotation. There are 2935 putative candidates for a total of 112 unknown identities and each identity has more than 3 putative candidates. Panels **a**–**j** show the improvement of ranking putative candidates. Panels **d**–**g** show the improvement of filtering putative candidates. Examples are given of some molecules ranking relatively poorly in OCMs 19–24 (**a**), some molecules ranking relatively poorly in OCMs 25–30 (**b**), and some molecules ranking almost uniformly across all OCMs (**c**) before calibration. The ranking of these molecules reached similar levels among all OCMs after calibration (**a**–**c**). Panels **h**, **i** show the differences in the number of correct candidates is exactly the Nth top candidate among 30 OCMs before (**h**) and after calibration (**i**). An example is given of using post–projection calibration method to improve the correct candidate ranking (**j**). Panel **d** shows the filtering error threshold for 30 OCMs. Panel **e** shows the true negative rate (TNR). Panel **f** shows the true positive rate (TPR). Panel **g** shows the accuracy of filtering putative candidates by RT error threshold.

Filtering and ranking results for the 2935 putative candidates are shown in Fig. 8 and Supplementary Data 14. First, we used an RT error threshold set at twice the RMSE_Ret_ for the corresponding OCM to sift through the candidate list (Fig. 8d). In each OCM, candidates with predicted RT and experimental RT (before calibration) or RePRT (after calibration) difference above this threshold were deemed negative, while those within the threshold were considered positive. Then, we determined the filtering accuracy, true–positive rate (TPR), and true–negative rate (TNR) after calculating the number of true positive (TP), false positive (FP), true negative (TN) and false negative (FN) candidates (see Methods for details on these calculations). We demonstrated that for OCMs 19–30, the filtering results after calibration (TNR = 57.3%–69.0%) were more accurate than those before calibration (TNR = 35.0%–51.1%, Fig. 8e). These defined error thresholds and quantitative filtering outcomes (Fig. 8d–f) underscore a notable enhancement in filtering efficacy when using post–projection calibration. The overall filtering accuracy was between 58.6% and 71.3% (Fig. 8g). It is noteworthy that after calibration, about 29% to 43% of false candidates in the OCM were accepted, and about 6% to 20% of true identities were incorrectly filtered for many reasons, including large prediction errors from the QSRR model and small changes in RT for those isomeric candidates using conventional LC methods.

Finally, we focused on the ability of post–projection calibration to rank putative candidates, i.e., the ranking results before and after calibration were compared. We demonstrated that about 57% of the correct identities showed improved rankings after calibration. These improvements were largely observed in OCMs that have a different mobile phase from the ICM 03p, especially for those identities with outgeneral retention behavior (Fig. 8a, b). Yet, identities with general retention behavior showed little improvement (Fig. 8c). Overall ranking results showed that the correct identities in OCMs 01–18 ranked better than in OCMs 19–30 before calibration (Fig. 8h), and that the correct identities ranked consistently across all OCMs after calibration (Fig. 8i). Specifically, before calibration, the number of true identities among the top 5 candidates (Ntop5) were ranged from 58 to 81 in OCMs 01–30 before calibration, while after calibration, it was in all cases above 76 (67.9% of all identities). Figure 8j shows an example of improved ranking with calibration in OCM 24. The correct identity, vardenafil, was ranked 2nd among 32 putative candidates using RePRT (15.8 min) to compare with predicted RT (15.5 min), while it dropped to 25th using experimental RT (10.2 min) for comparison.

## Discussion

In this work, we developed a generic and accurate method, namely, post–projection calibration, to support RT prediction in RPLC. This method builds on the direct projection of RTs between two different CMs and introduces RePRT instead of traditional experimental RT for comparison with projected RT. Although several methods have been developed to predict RT across different CMs, the post–projection calibration reduces prediction errors introduced by inconsistencies in RT profile between two CMs. That is, with RePRT, predictions with large errors in such CMs are calibrated and accepted, whereas in general experimental–experimental projections, e.g., PredRet^8,30^, they are usually discarded due to having large prediction intervals. One possible concern is the inconsistency in RT profile between ReICM and ICM. Our results showed that using inappropriate ReICM, most molecules have < 1% errors of elution time between RePRT and projected RT, but some molecules showed larger errors up to 23.9%. Therefore, we developed a RT database for heterogeneous molecules for a wide range of reversed–phase liquid chromatographic methods (i.e., MCMRT), and selected molecules with diverse retention behaviors as calibrants. These calibrants enables to select the most appropriate ReICM based on their RT profiles in ReICM and ICM. We are also working on collaborating with other laboratories to determination the RTs for calibrants and other small molecules in different CMs, to expand the MCMRT database and further validate the method performance. We believe that with the widespread use of calibrants across laboratories, the availability of RT datasets will largely improve.

The post–projection calibration approach enables the community to utilize experimental RT values from publicly available datasets for model training, and accurately share both experimental and predicted RT values onto a given CM. The relative median error for predicted RTs was between 4.6% and 12.2%. Comparing the accuracy of this approach with other published prediction methods is challenging, because molecules in the validation set are often inconsistent and prediction errors with the same statistics are not always reported. For similar published methods, e.g., CALLC^31^, MultiConditionRT^22^, RTI system^9^, and PredRet (predicted–experimental projections)^18–21^, median prediction errors of about 0.1–3 min were typically observed, corresponding to relative errors of ~6–20%. In that sense, our method for predicted RTs showed better performance. However, some limitations in projecting predicted RTs are still presented. For example, most of molecules can be accurately predicted from their structure to their RT, but some may have larger error in their predicted RT due to the absence of their similar structures in the training set. Currently, post–projection calibration can only calibrate the prediction error caused by LC setups. The use of large retention database for structure–based RT modeling may help to address this challenge. In addition, although post–projection calibration has made effective improvements up to ~20% filtering accuracy, the identification of isomers remains a challenge due to the limit separation of LC methods.

## Methods

### RT acquisition for the MCMRT database

The pure standard materials for the 343 molecules were analyzed on a Vanquish UHPLC System (Thermo Fisher Scientific, USA) coupled to an Orbitrap Q–Exactive Plus mass spectrometer (Thermo Fisher Scientific, USA) using 30 different CMs. Each CM–dependent dataset was acquired within one day, with three repetitions. These CMs covered six C_18_ columns with different manufacturers, column lengths (50–150 mm), diameters (2.1–4.6 mm) and particle sizes (1.7–5 μm). The gradients consist of single and multi–slopes with isocratic or gradient flow rates of 0.2 to 0.5 mL/min. The total running times ranged from 10 to 100 min. Among these CMs, 18 used water/methanol (90:10, v/v) as mobile phase A, 12 used water as mobile phase A, 24 used methanol as mobile phase B, and 6 used acetonitrile as mobile phase B. Different additives were considered in these mobile phases, such as 0.01% formic acid with 5 mM ammonium formate (A1B1), 0.1% formic acid with 4 mM ammonium formate (A5B5), 0.1% formic acid (A2B2), 5 mM ammonium formate (A4B4), and 5 mM ammonium acetate (A3B3). In addition, column temperatures ranging from 30 °C to 45 °C were included. A full description of LC setups for each CM can be found in Supplementary Data 2. All analyses were performed in positive and negative ionization mode using a full mass scan. The parameters were as follows: two scan ranges for 80–400 Da/350–1600 Da; resolution = 70,000; AGC target = 1e6; maximum injection time = 100 ms; sheath gas = 40; aux gas = 8; sweep gas = 1; spray voltage = 2.5 kV; heater Temp = 350 °C; Capillary Temp = 250 °C; RF–Lens = 60.

### QSRR model construction and parameters

The model construction and hyperparameter optimization was performed in MATLAB R2021b. A set of 1820 chemicals of emerging contaminants with known RT values on CM 03p was collected from the report by Aalizadeh et al. ^9^. From all the molecules in CM 03p (except for 154 that overlapped with our 343 molecules), 75% of them (*N* = 1258) were randomly selected and used as a training set, whereas the remaining molecules including the overlapping molecules (*N* = 562) were used as validation set. Their molecular structure data (with extension *.mol*) were collected from the PubChem database (https://pubchem.ncbi.nlm.nih.gov/), and a total of 1444 2D–molecular descriptors were calculated for each compound using PaDel-descriptor^39^. An artificial neural network (ANN) based on multilayer perceptron was used to develop the QSRR model. The perceptron consisted of 1444 artificial neurons in the input layer, 10 artificial neurons in the hidden layer, and 1 neuron in the output layer. The Bayesian Regularization Backpropagation method was used to train the network, and the learning was completed in 58 epochs. ANN analysis was performed on the training and validation sets by an iterative minimization program to optimize parameters. This model was used to predict the RT of 343 molecules in the MCMRT database and 2935 putative candidates for 112 unknown identities observed on 30 OCMs (see section “Retention time projection”). From the 343 molecules, 335 with observed experimental RT on CM 03 were used as the external set to validate the generalization ability of the learned ANN via predicted–experimental projection. The predicted RTs of these molecules were projected onto CM 03 and compared with their experimental RTs.

### Retention time projection

The projection of RT values from one CM onto another is performed in pairs by MATLAB R2021b. For each pair of ICM (or ReICM) and OCM, experimental RTs of calibrants are used to build a gaussian process (GP) based nonlinear regression model between RTs in the two CMs. The basis and kernel functions in GP were set as constant and rational quadratic, respectively. The hyperparameters were optimized with the quasi–newton method. This model allowed adjusting all the experimental and predicted RTs via experimental/predicted–experimental projections to make them comparable to the OCM.

### Post–projection calibration for experimental RT

All 30 CMs in MCMRT were used as ICMs, and for each ICM, an appropriate ReICM was selected from the remaining 29 CMs, followed by using the remaining 28 CMs as their OCMs, respectively. To select the most appropriate calibrants, we used five different sets of calibrants to perform post–projection calibration, including projecting all experimental RTs from ICM to OCM and calibrating all projected RTs in each OCM. Specifically, the experimental RTs of calibrants in each pair ICM and OCM were used to train a main projection model, to derive the projected RT in OCM for all molecules in MCMRT. The projected and experimental RTs in each OCM were compared to test the performance of experimental–experimental projections. Then, the experimental RTs of calibrants in each pair of ReICM and OCM were used to train a ReProjection model, to derive the RePRT in OCM for all molecules in MCMRT. The projected RT and RePRT in each OCM were compared to test the performance of post–projection calibration for experimental RT.

### Post–projection calibration for predicted RT

CM 03p and CM 07 were used as ICM and ReICM, respectively. The remaining 29 CMs in MCMRT were used as OCMs, respectively. Thirty-five calibrants in Set B were used to train projection and ReProjection models, of which 26 calibrants used experimental RT, while the remaining 9 calibrants used predicted RT due to the lack of experimental RT on CM 03p. For ReProjection models, the experimental RTs of the 33 calibrants were used. The predicted RT for all the molecules in MCMRT and 2935 putative candidates was projected from ICM 03p to 29 OCMs using projection models, and the RePRT was derived from their experimental RT on CM 07 using ReProjection models.

### Impact of prediction error on post–projection calibration

To assess whether the prediction error of molecules affects the performance of post-projection calibration, we compared the predicted–experimental projections before and after calibration in different OCM groups within the external set. Molecules in the validation set were divided into four groups based on their projection error in CM 03. Among them, group 1 contains 209 molecules with an error of less than 1 min, group 2 contains 68 molecules with an error of between 1 and 2 min, group 3 contains 44 molecules with an error of between 2 and 4 min, and group 4 contains 14 molecules with an error of greater than 4 min. The T-test was used to evaluate statistical significance, with a significance level set at 95%. Exact P-values are described in the Results section.

### Filtering-associated parameters

For each filtering threshold, the number of true negatives (TN), true positives (TP), false negatives (FN) and false positives (FP) were calculated. TN was the number of false identities with an RT error above the error threshold, TP was the number of true identities with an RT error below the error threshold, FN was the number of true identities with an RT error above the error threshold, and FP was the number of false identities with an RT error below the error threshold. The true positive rate (TPR) was calculated as TP/(TP + FN), the false positive rate (FPR) was calculated as FP/(FP + TN), and the filtering accuracy was calculated as (TP + TN)/(TP + FP + TN + FN).

### Supplementary information

Supplementary Information

Description of Additional Supplementary Files

Supplementary Data 1-14

Supplementary Data 15

## Data Availability

The MCMRT database, including chemical information, LC conditions, and experimental RT data, is available in Supplementary Data 1–14. This section also includes both the raw and statistical analyses of the experimental–experimental and predicted–experimental projections before and after calibration. Moreover, the numerical source data for Figs. 2–8, which initially consisted of more than 10 Excel files, has been consolidated into a single Excel file (as Supplementary Data 15), with distinct tabs allocated for each specific dataset. The data can be freely accessed on GitHub at https://github.com/Yanzi-Zhang-oss/Post-projection-calibration-of-retention-time-across-liquid-chromatography-setups. Any additional data can be obtained from the corresponding author upon reasonable request.

## Code availability

The source code of RT projection, QSRR modeling and SOM clustering algorithm was provided in GitHub (https://github.com/Yanzi-Zhang-oss/Post-projection-calibration-of-retention-time-across-liquid-chromatography-setups).
