# Supplementary material for: Generic and accurate prediction of retention times in liquid chromatography by post–projection calibration
Source: Commun Chem. 2024 Mar 8;7:54. doi: 10.1038/s42004-024-01135-0 (PMC10923921; doi:10.1038/s42004-024-01135-0)
Supplement: Supplementary file 1 — Supplementary Information [file 42004_2024_1135_MOESM1_ESM.pdf]

Generic and accurate prediction of retention times in liquid chromatography by post-projection calibration

Supplementary information for the paper entitled Generic and accurate prediction of retention times in liquid chromatography by post-projection calibration. Supplementary discussions show the results of retention behavior classification and calibrants selection. Figure S1 shows the relationship between the experimental RTs of all molecules for each pair of CMs in MCMRT. Figure S2 through S6 compare the RT profiles demonstrated by all molecules in MCMRT and five different sets of calibrants. Figure S7 compare the square of the correlation coefficient (r2) between the experimental RTs of 343 molecules in ICM and OCM for different OCM groups. Figure S8 show the relation between the root mean square error relative to elution time square ($RMSE_{Ret}$) and the correlation coefficient (r2) between the experimental RTs of calibrants molecules in ICM and OCM.

Supplementary discussions

Retention behavior classification. Thirty RT datasets in MCMRT were analyzed for 330 overlapping molecules using self–organizing mapping (SOM) clustering algorithm, to characterize their retention behavior. These molecules were classified into 25 groups, each of which exhibited a specific RT distribution and similar retention behavior with different LC setups (Supplementary Data 3 and Fig. 3). Specifically, the non–retained molecules were included in groups 1–2, while in CM without acidic additives (CMs 25–30), molecules in group 2 exhibited stronger retention than those in group 1 (Figs. 3a–b). The molecules in groups 3–5 had relatively weak retention in 30 CMs (Figs. 3c–e), while the molecules in groups 12, 16, 21–24 had stronger retention than those in other groups (Fig. 3). For molecules in groups 1, 3, 5, and 13, there was no large difference in RT among CMs 03, 19, 20 and 30 (these CMs only differed in the composition of the mobile phases). However, the retention of molecules in the remaining 21 groups was affected by additives. For example, for molecules in group 16, the RT in CM 20 was 23.5–30.2% larger than that in CM 30 (Fig. 3p), while for molecules in group 19, it was 18.3–35.4% smaller than that in CM 30 (Fig. 3s). The molecules in groups 7, 9, 11 and 14 exhibit similar retention behaviors, representing a general variation in RT between different LC setups (Figs. 3g, i, k, n). Large differences were observed in the retention behavior for molecules in the remaining groups. For example, for molecules in group 15, the RT in CM 24 was 2.0–7.1 min larger than that in CM 22 (Fig. 3o), while for molecules in group 18, it was -0.7–4.7 smaller than that in CM 22 (Fig. 3r), and for molecules in other groups, the RT in these two CMs was almost identical. These results demonstrated that although in most cases many molecules exhibit consistent RT changes between two different CMs, there can still be several outgeneral changes for a considerable number of molecules.

Nevertheless, these changes had a negative impact on the conservation of molecular elution order. We thus used the square of the correlation coefficient (r2) between the experimental RTs of 343 molecules in two different CMs to determine the CM similarity in molecular elution order, and demonstrated that the elution order of molecules can be largely different even between very same types of LC columns (Fig. S1). Specifically, two different CMs with similar C_{18} column specifications, similar elution times, and the same mobile phase composition had highly similar elution order, with r2 ranging from 0.990 to 0.998. The difference in C_{18} column specifications slightly decreased the CM similarity, with r2 as low as 0.953. In addition, the larger the difference in running time between CMs, the lower r2. For example, the r2 between CM 01 (10 min) and CM 06 (21 min) was 0.959, while the r2 between CM 01 and CM 16 (100 min) was 0.934. The mobile phase composition had the greatest negative impact on the elution order, as more molecules exhibited outgeneral changes in RT.

This was because additives changed the pH value and ionic strength of solvents, thereby altering the retention behavior of many molecules through protonation, deprotonation or ionic interaction. Specifically, similar mobile phases yielded the r2 of 0.921–0.993. The r2 values ranged from 0.789 to 0.947 between mixed additives and other additives (e.g., semi–mixed, acidic and ammonium additives). The lowest r2 values were often observed between ammonium and acidic additives, ranging from 0.689 to 0.847. Taken together, the larger and more differences in LC setups, the lower the CM similarity in molecular elution order. Examples are given of four representative relationships between experimental RTs in two CMs with different LC setups, including that in two CMs with identical mobile phase compositions (r2 = 0.991, Fig. 4a), that in two CMs with similar mobile phase compositions (r2 = 0.940, Fig. 4b), that in two CMs with dissimilar mobile phase compositions (r2 = 0.913, Fig. 4c), and that in two CMs with very different mobile phase compositions (r2 = 0.698, Fig. 4d).

Selection and characterization of calibrants. Five different sets of calibrants were selected from 330 overlapping molecules in MCMRT, namely set A containing 27 molecules, set B containing 35 molecules, set C containing 60 molecules, set D containing 72 molecules, and set E containing 39 molecules. The aim was to select the most appropriate set of calibrants for reliable post–projection calibrations. Sets A, B, C and D all covered 25 retention behaviors, and as the number of calibrants increased from 27 to 72, more molecules with general retention behavior were included. In addition, the molecules with outgeneral retention behavior were more included in set D than in set C. The calibrants in set E were specially customized and only covered 17 retention behaviors. The molecules in groups 8, 10, 15, 17, 18, 19, 23 and 25 were not included in this set.

The correlation between experimental RTs in two different CMs was demonstrated using the five sets of calibrants and the 330 overlapping molecules in MCMRT, respectively. Specifically, the r2 between experimental RTs for each pair of CMs ranged from 0.688 to 0.999 when using the 330 overlapping molecules, while when using the calibrants in sets A, B, C, D and E, it ranged from 0.701 to 0.999, 0.678 to 0.998 0.728 to 0.999, 0.681 to 0.999 and 0.842 to 0.999, respectively. For sets A, B, C and D, the r2 difference between calibrants and 330 molecules was in all cases below 0.07, while for set E it can be as high as 0.16. In addition, the calibrants in sets A, B, C, and D not only covered the entire RT range of each CM, but also exhibited RT profile highly similar to the 330 molecules (Figs. S2–5). Although the calibrants in set E covered the entire RT range, they exhibited limited representativeness for molecules with outgeneral retention behavior (Fig. S6). Collectively, calibrant sets A, B, C and D can account for the effect of LC setups on molecular elution order and demonstrate the overall elution pattern of a CM.

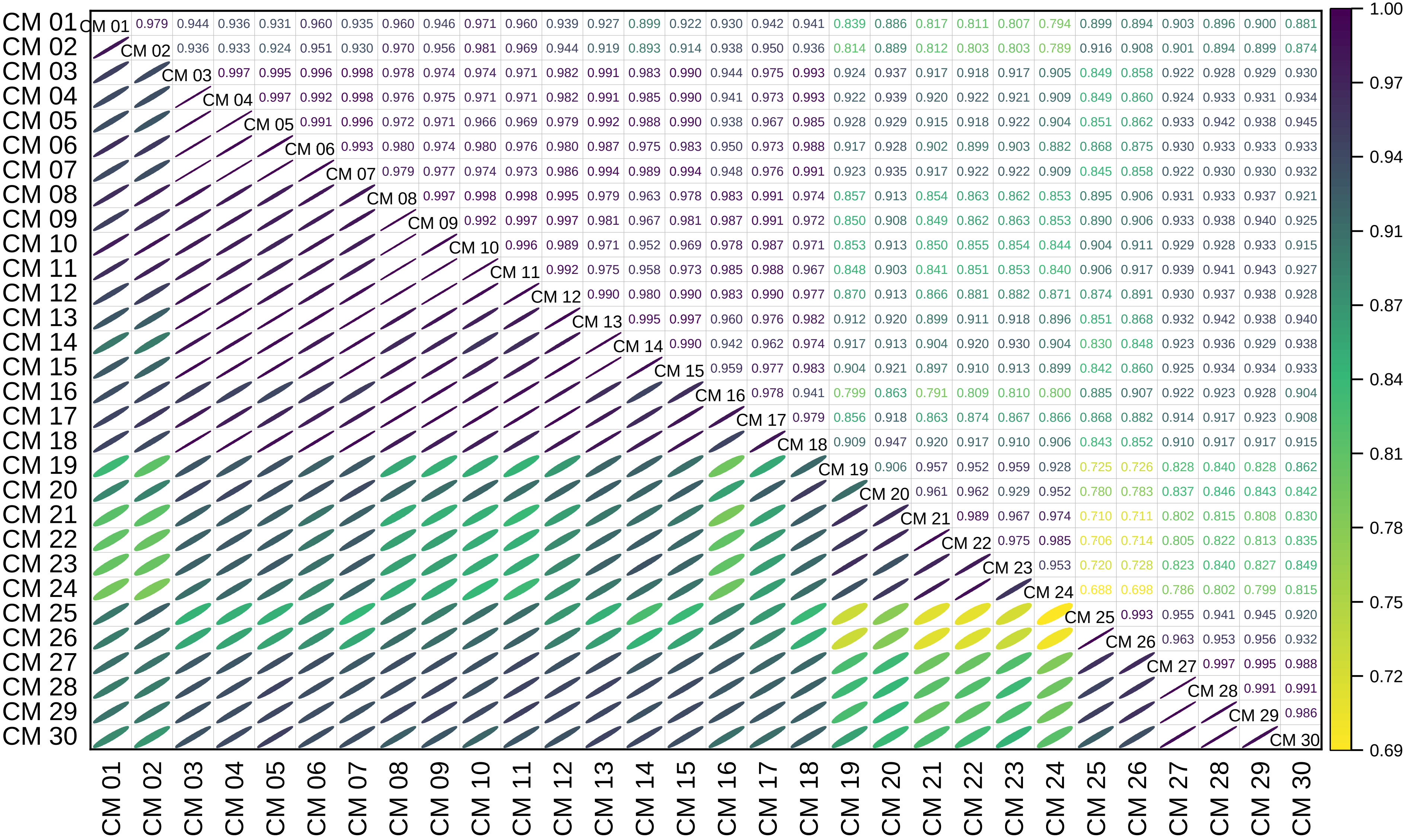

Fig. S1 The relationship (r2) between the experimental RTs of all molecules for each pair of CMs in MCMRT.

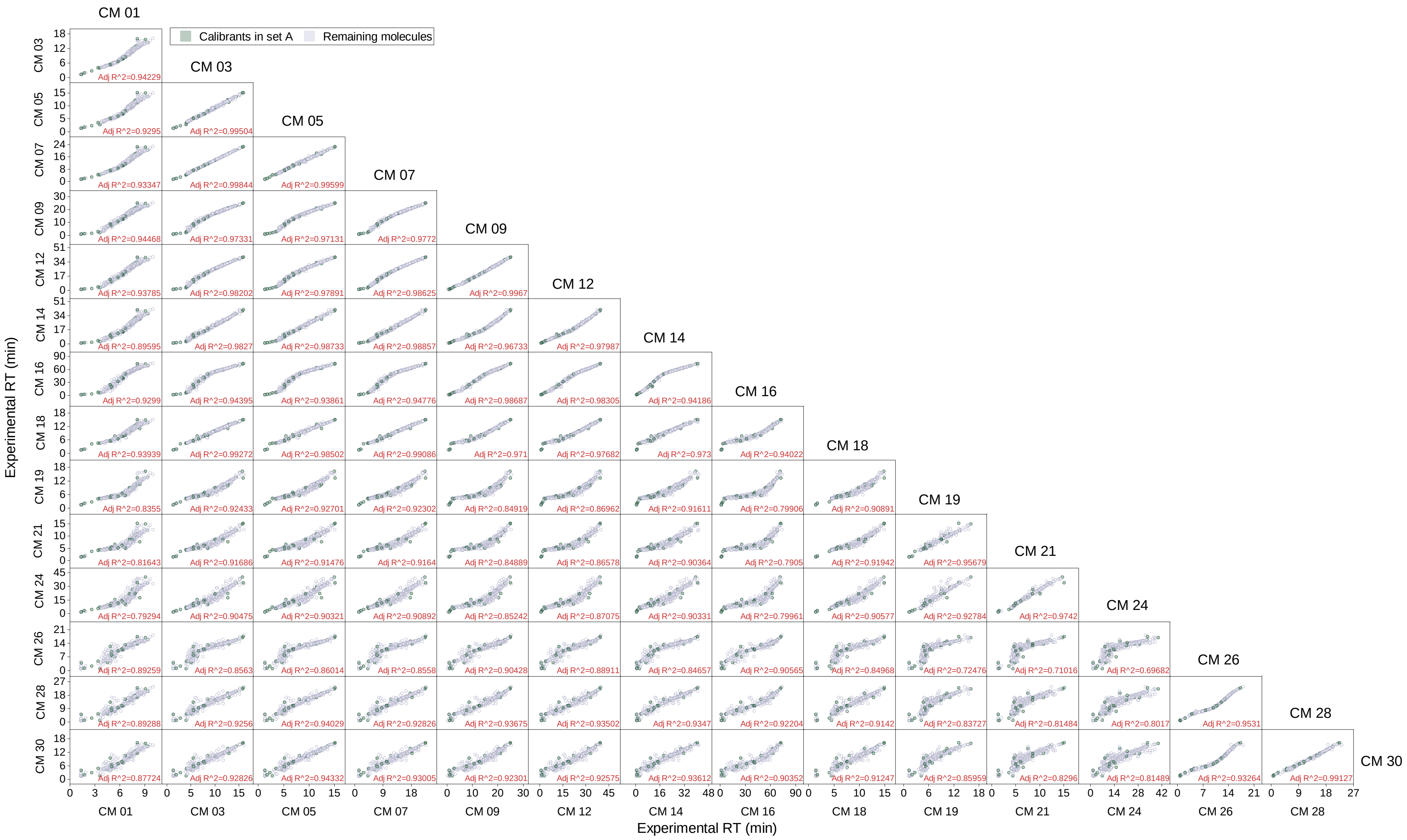

Fig. S2 Comparison of experimental RTs with different CMs for all molecules in MCMRT and 27 calibrants in set A.

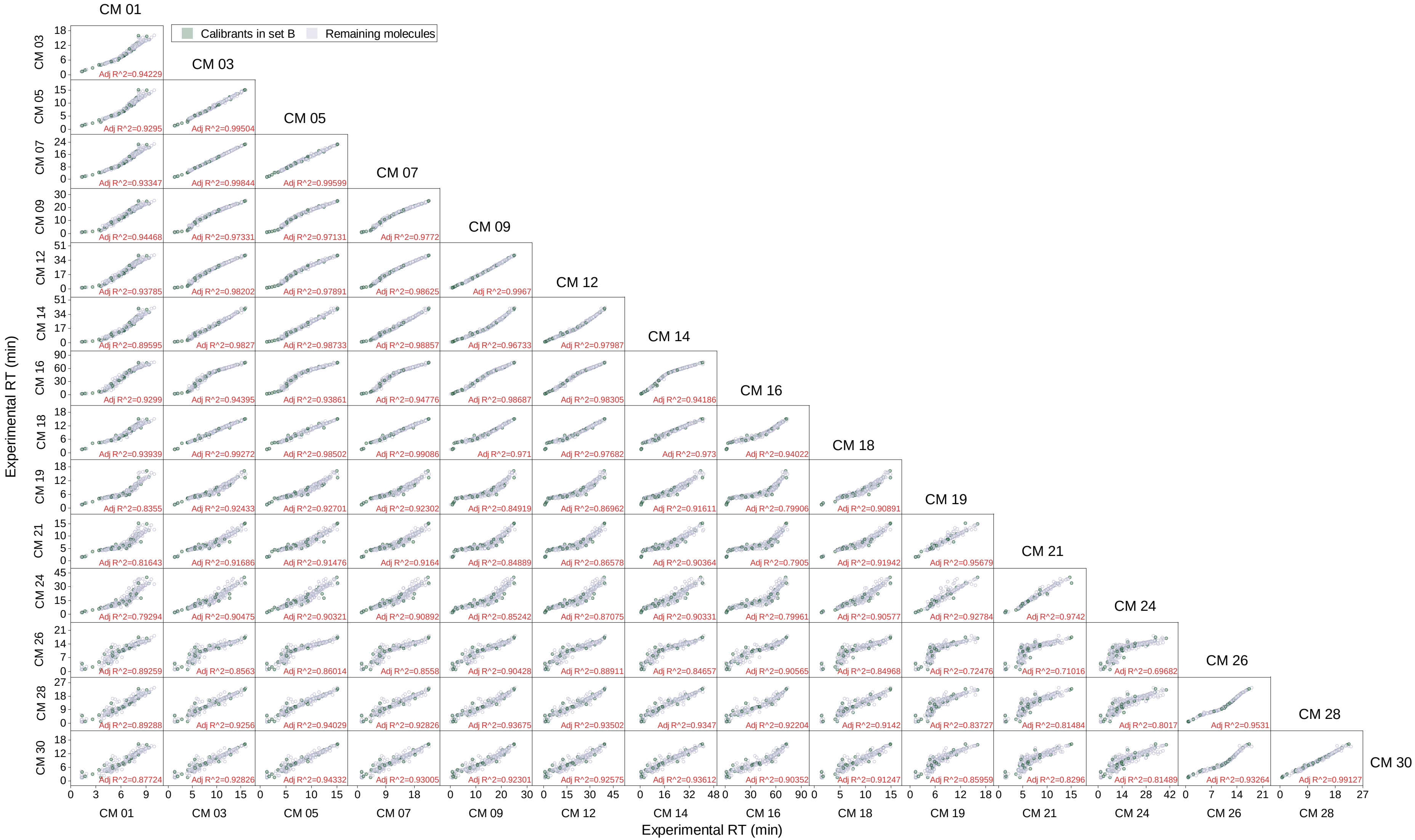

Fig. S3 Comparison of experimental RTs with different CMs for all molecules in MCMRT and 35 calibrants in set B.

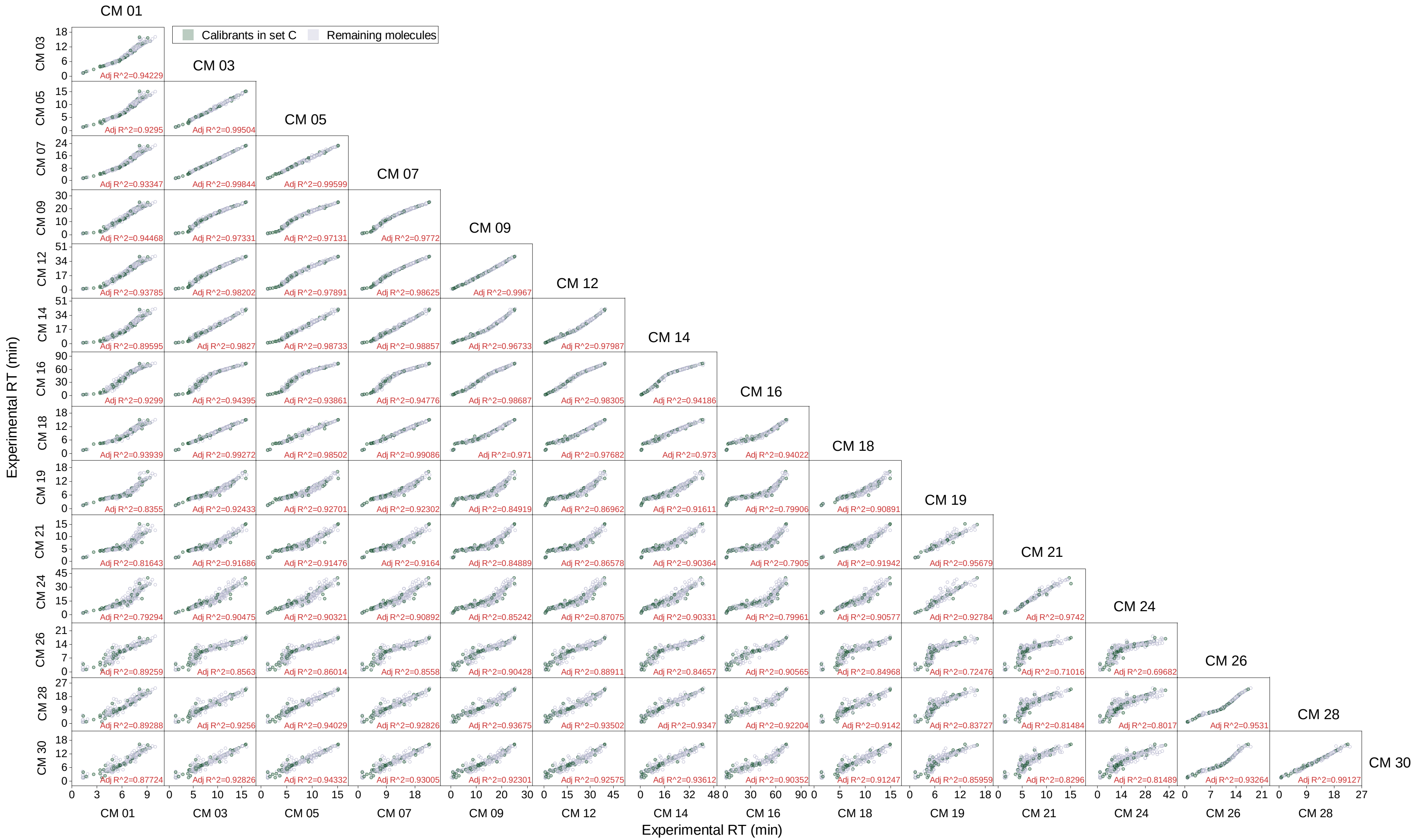

Fig. S4 Comparison of experimental RTs with different CMs for all molecules in MCMRT and 60 calibrants in set C.

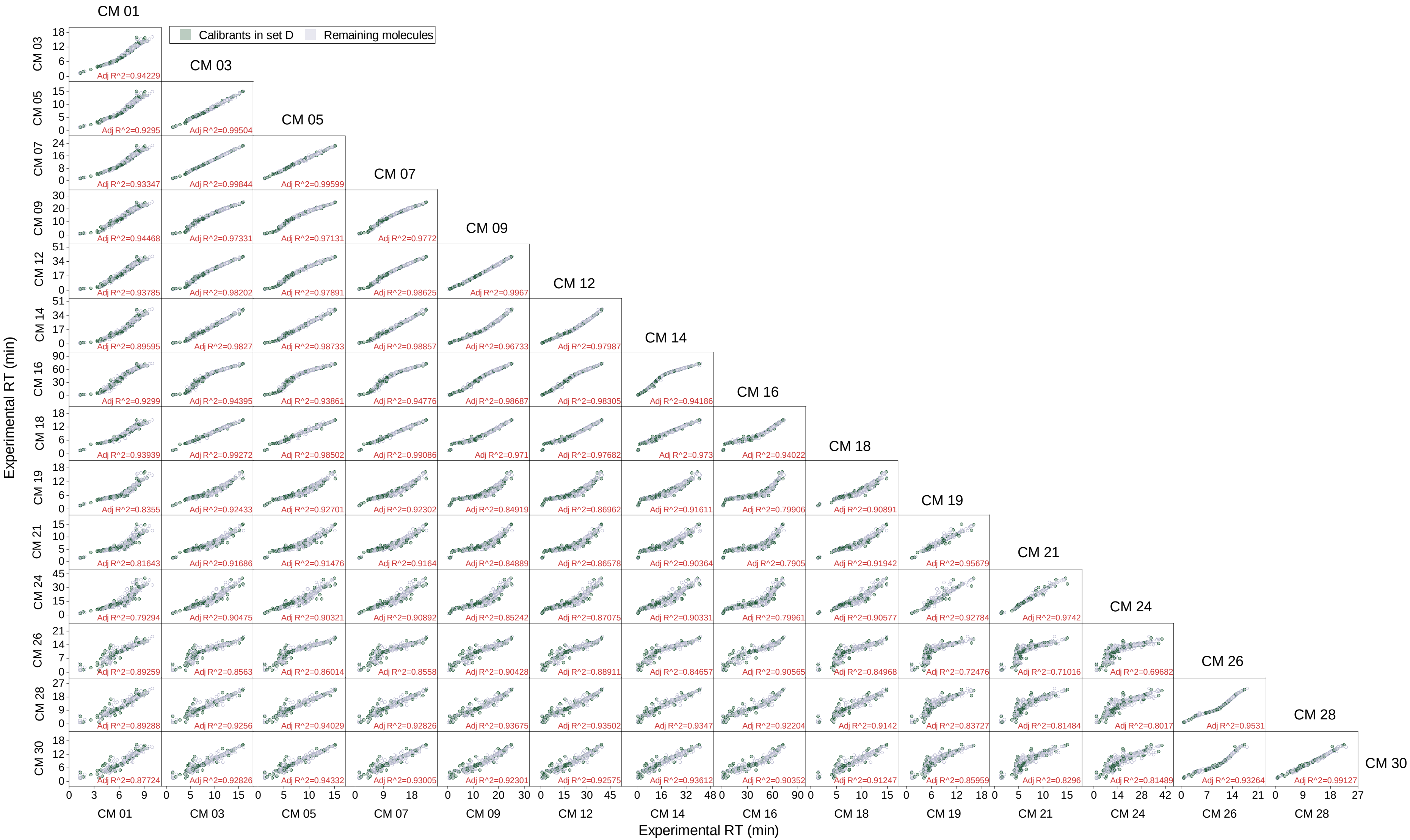

Fig. S5 Comparison of experimental RTs with different CMs for all molecules in MCMRT and 72 calibrants in set D

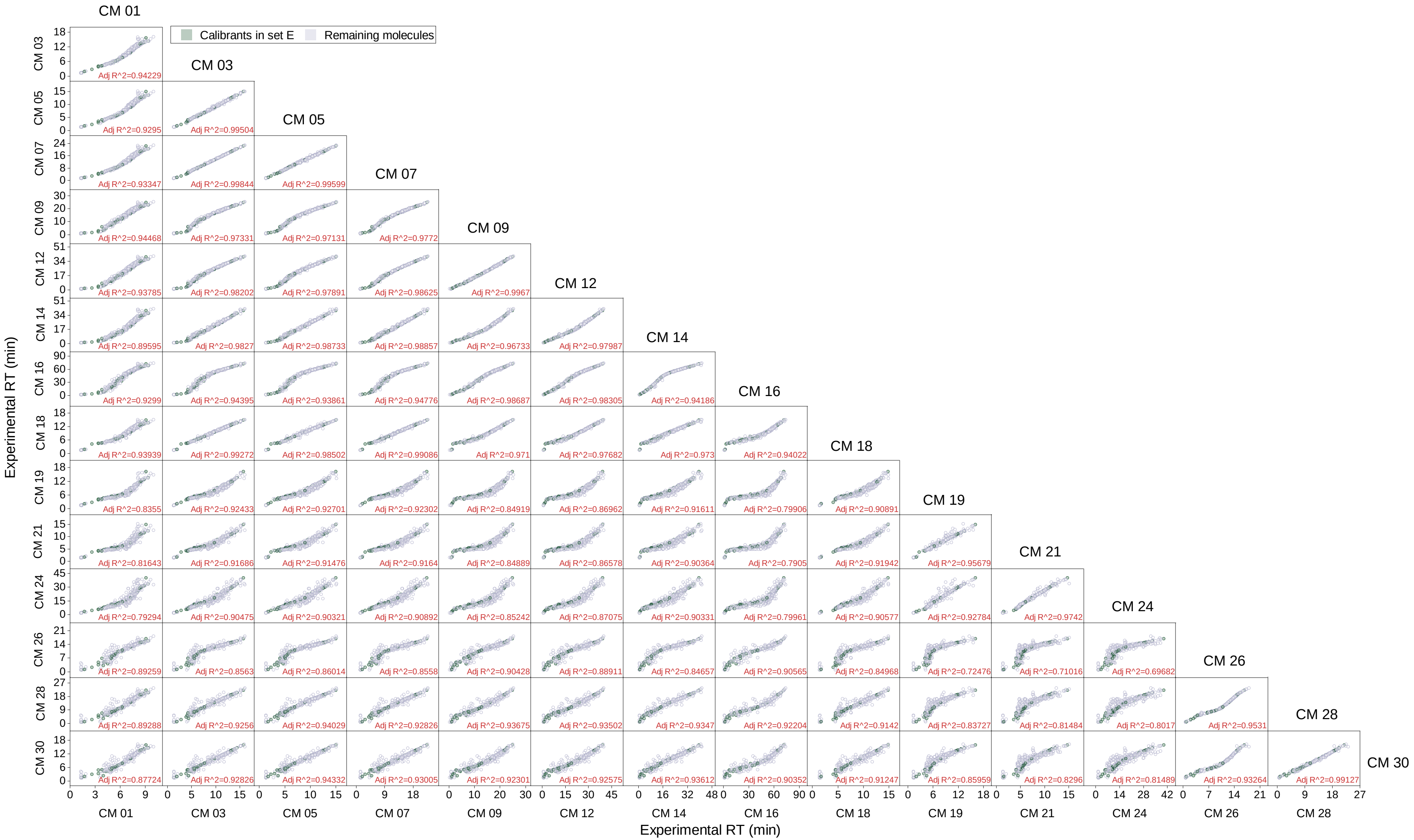

Fig. S6 Comparison of experimental RTs with different CMs for all molecules in MCMRT and 39 calibrants in set E.

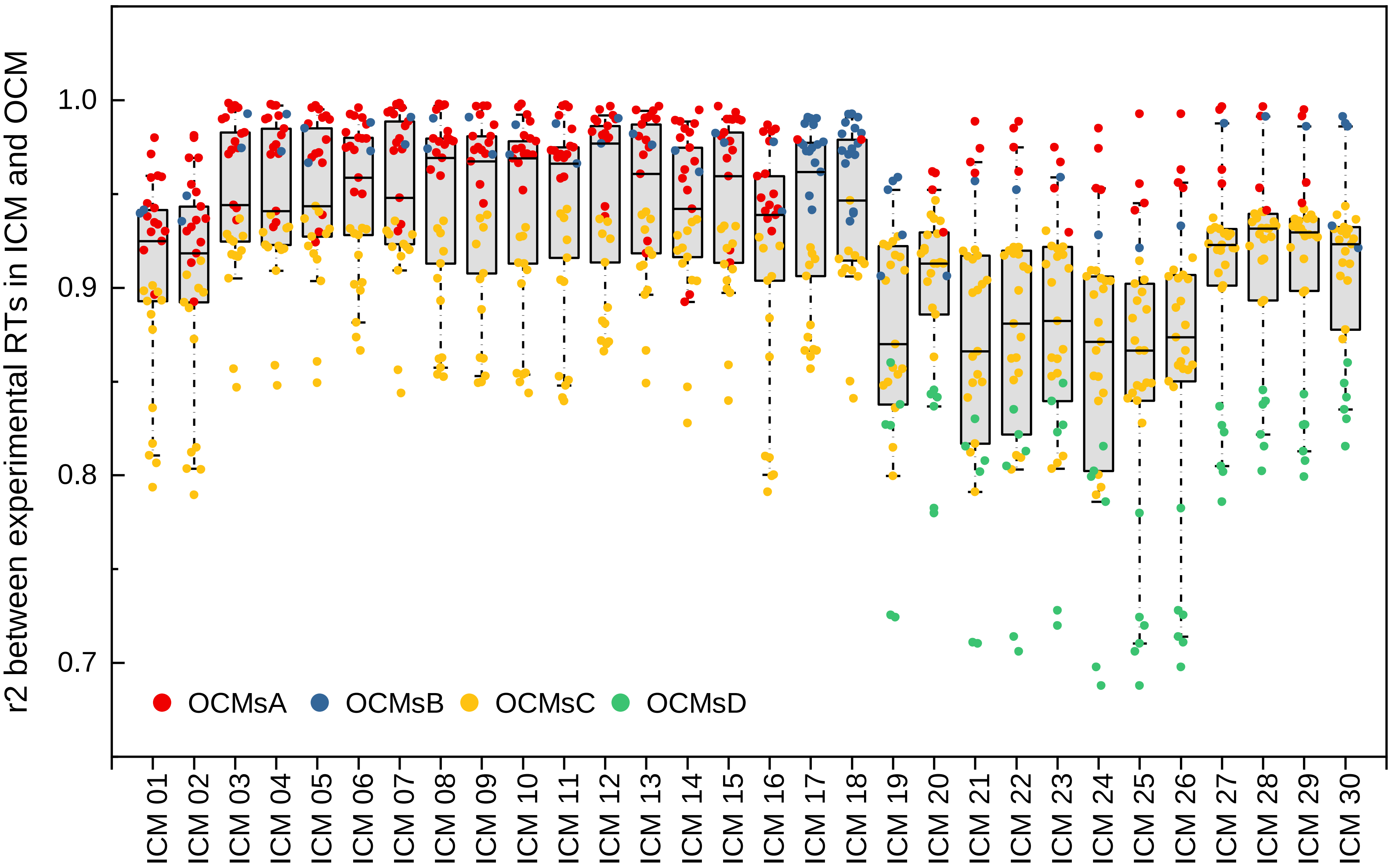

Fig. S7 The square of the correlation coefficient (r2) between the experimental RTs of 343 molecules in ICM and OCM.

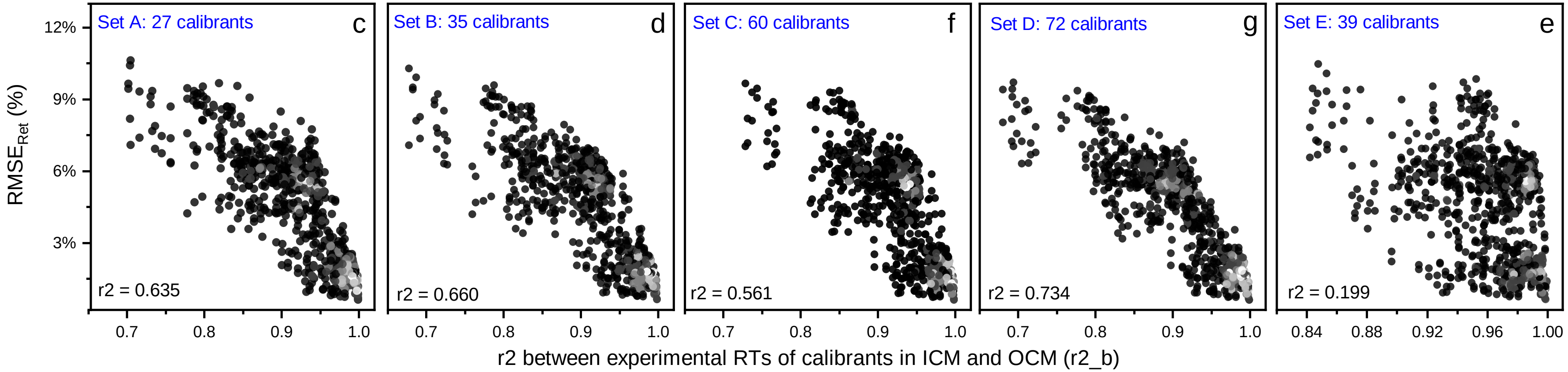

Fig. S8 The relation between the root mean square error relative to elution time square (RMSE$_{Ret}$) and the correlation coefficient (r2) between the experimental RTs of calibrants molecules in ICM and OCM.
