# Supplementary material for: Generic and accurate prediction of retention times in liquid chromatography by post–projection calibration
Source: Commun Chem. 2024 Mar 8;7:54. doi: 10.1038/s42004-024-01135-0 (PMC10923921; doi:10.1038/s42004-024-01135-0)
Supplement: Supplementary file 2 — Description of Additional Supplementary Files [file 42004_2024_1135_MOESM2_ESM.pdf]

Description of Additional Supplementary Files

File name: Supplementary Data 1

Description: outlines the specifics of 343 molecules, including names, chemical identifiers, molecular formula, weight, and classification.

File name: Supplementary Data 2

Description: elaborates on the instrumental and chromatographic setups for 30 Chromatographic Methods (CMs).

File name: Supplementary Data 3

Description: compiles the experimental retention times of the 343 molecules across the 30 CMs.

File name: Supplementary Data 4

Description: reveals squared correlation coefficients for retention times comparing all Input Chromatographic Method (ICM) and Output Chromatographic Method (OCM) pairs, covering molecules and calibrants in Sets A to E

File name: Supplementary Data 5

Description: details the molecular profiles within each calibrant set.

File name: Supplementary Data 6

Description: delivers insights into all OCM groupings.

File name: Supplementary Data 7

Description: present statistical analyses of experimental projections for each ICM and OCM pair, before calibration with Set B calibrants.

File name: Supplementary Data 8

Description: present statistical analyses of experimental projections for each ICM and OCM pair, after calibration with Set B calibrants.

File name: Supplementary Data 9

Description: examines the statistical outcomes of experimental projections within each OCM, before and after calibration, using Set B calibrants, with five different CMs serving as Reference Input Chromatographic Methods (ReICMs) for ICM 10.

File name: Supplementary Data 10

Description: showcase statistical analyses of projections within each ICM and OCM pair, before and after calibration, utilizing calibrants from five distinct sets.

File name: Supplementary Data 11

Description: showcase statistical analyses of projections within each ICM and OCM pair, before and after calibration, utilizing calibrants from five distinct sets.

File name: Supplementary Data 12

Description: llustrates the predictive accuracies of the QSRR model for various datasets.

File name: Supplementary Data 13

Description: focus on the statistical analyses of predicted versus experimental projections within each OCM, before calibration using Set B calibrants, and the evaluation of 2935 potential candidates.

File name: Supplementary Data 14

Description: focus on the statistical analyses of predicted versus experimental projections within each OCM, after calibration using Set B calibrants, and the evaluation of 2935 potential candidates.

File name: Supplementary Data 15

Description: The raw data underlying the figures comprising original datasets for Figures 2 through 6.
